# Lower Beta: A Central Coordinator of Temporal Prediction in Multimodal Speech

**DOI:** 10.3389/fnhum.2018.00434

**Published:** 2018-10-24

**Authors:** Emmanuel Biau, Sonja A. Kotz

**Affiliations:** ^1^Basic and Applied Neuro Dynamics Laboratory, Department of Neuropsychology and Psychopharmacology, Faculty of Psychology and Neuroscience, University of Maastricht, Maastricht, Netherlands; ^2^Department of Neuropsychology, Max Planck Institute for Human Cognitive and Brain Sciences, Leipzig, Germany

**Keywords:** temporal predictions, beta oscillations, multimodal speech perception, prosody, biological motion

## Abstract

How the brain decomposes and integrates information in multimodal speech perception is linked to oscillatory dynamics. However, how speech takes advantage of redundancy between different sensory modalities, and how this translates into specific oscillatory patterns remains unclear. We address the role of lower beta activity (~20 Hz), generally associated with motor functions, as an amodal central coordinator that receives bottom-up delta-theta copies from specific sensory areas and generate top-down temporal predictions for auditory entrainment. Dissociating temporal prediction from entrainment may explain how and why visual input benefits speech processing rather than adding cognitive load in multimodal speech perception. On the one hand, body movements convey prosodic and syllabic features at delta and theta rates (i.e., 1–3 Hz and 4–7 Hz). On the other hand, the natural precedence of visual input before auditory onsets may prepare the brain to anticipate and facilitate the integration of auditory delta-theta copies of the prosodic-syllabic structure. Here, we identify three fundamental criteria based on recent evidence and hypotheses, which support the notion that lower motor beta frequency may play a central and generic role in temporal prediction during speech perception. First, beta activity must respond to rhythmic stimulation across modalities. Second, beta power must respond to biological motion and speech-related movements conveying temporal information in multimodal speech processing. Third, temporal prediction may recruit a communication loop between motor and primary auditory cortices (PACs) via delta-to-beta cross-frequency coupling. We discuss evidence related to each criterion and extend these concepts to a beta-motivated framework of multimodal speech processing.

Continuous speech perception shapes spontaneous oscillatory activity in neuron ensembles, ensuring coordinated signal processing between specialized areas for successful comprehension. It has been now established that the sampling of sensory input temporal structure relies on the brain’s low frequency entrainment (1–8 Hz) to relevant rhythmic features occurring at slow time scales, and present in both auditory and visual modalities (Lakatos et al., 2008; Arnal and Giraud, 2012; Park et al., 2015). How does the brain integrate together speech structural information conveyed in two sensory modalities and processed separately in their corresponding cortical areas? In the present review, we discuss the role of lower beta oscillations (~20 Hz), originating from motor cortex and generally associated with motor functions (Engel and Fries, 2010; Press et al., 2011; Di Nota et al., 2017), as a potential amodal coordinator that integrates structural information of the speech signal extracted in specialized areas via entrainment mechanism. Correct multimodal integration of the continuous speech structure encoded in low-frequency patterns is crucial, as it would allow the brain to benefit from recurrent information across modalities, and in return generate stronger temporal predictions to ease the sensory sampling of incoming input.

Prosodic modulations and the syllabic chain (see “Glossary” section) impose peaks of energy fluctuations at delta (1–3 Hz) and theta (4–8 Hz) rates in the speech envelope. These significant fluctuations of amplitude serve as relevant anchors for sensory areas to synchronize with incoming signal and drive low-level sensory processing. In the primary auditory cortex A1/A2, previous magnetoencephalography/electroencephalography (MEG/EEG) studies show that this entrainment is typically reflected by the synchronization of an optimal delta-theta phase and power of responding neuron ensembles with the onsets of prosodic and syllabic events occurring in speech. For instance, Gross et al. (2013) investigated how low-frequency brain’s oscillations in auditory cortex encode the continuous speech signal. They presented long, spoken segments of continuous stories to participants while recording their MEG signal. In comparing oscillatory responses in the brain during normal speech perception to the same stories presented backward, they reported a significant phase alignment between low-frequency components of the speech envelope and brain activity in the delta (1–3 Hz) and theta (3–7 Hz) bands. These results confirmed that low-frequency rhythmic features in the speech envelope aligned with endogenous low-frequency cortical activities in auditory areas. Additionally, Gross et al. (2013) reported that delta and theta alignment depicted distinct lateralization patterns, suggesting a functional distinction between the two frequency bands. A right lateralization was found in the frontal and temporal areas for delta entrainment, which could reflect prosodic analysis engagement (see for example Bourguignon et al., 2013). In contrast, a right lateralization was found only in the auditory cortex for theta activity, supporting its role in syllabic processing (see for example Luo and Poeppel, 2007; see glossary for an extended definition of entrainment mechanism; for further reading on entrainment in auditory modality, see Ahissar et al., 2001; Luo and Poeppel, 2007; Abrams et al., 2008; Lakatos et al., 2008; Schroeder et al., 2008; Nourski et al., 2009; Schroeder and Lakatos, 2009a; Arnal and Giraud, 2012; Giraud and Poeppel, 2012; Peelle and Davis, 2012; Park et al., 2015; Zoefel and VanRullen, 2016; Ding et al., 2016).

However, speech is often multimodal with the onsets of the articulatory movements preceding the correspondent auditory onsets with a stable temporal delay (Pilling, 2009; Vroomen and Stekelenburg, 2010; Baart et al., 2014). This temporal correspondence between two modalities (i.e., visual and auditory) allows the brain to generate inferences based on the leading modality such as to predict timing (“when”) and content (“what”) of the upcoming auditory input to create a unified percept (van Wassenhove et al., 2005; Stekelenburg and Vroomen, 2007; Peelle and Sommers, 2015). For instance in speech perception, lip movements might improve perceptual sensitivity to corresponding auditory information by means of a generic temporal prediction mechanism and top-down modulations to primary auditory cortices (PACs). At the neural level, visual information facilitates both temporal and content predictions as demonstrated by differential latency and amplitude reductions of N1/P2 auditory evoked potentials in the EEG signal (van Wassenhove et al., 2005). While the latency reduction of N1/P2 increased with the saliency of lip movements (i.e., pa > ta > ka) in audiovisual as compared to auditory condition, the amplitude reduction was equivalent across syllables and independent of visual saliency. Further, Arnal et al. (2009) also established that latency reductions of auditory evoked response (i.e., M100 component) were stronger for syllables produced by large and unambiguous mouth movements than produced with ambiguous facial movement, suggesting a role of visual information not only in temporal prediction but also in content predictions. These results suggest that the temporal alignment of corresponding visual and auditory information already facilitates speech processing, probably by focusing attention of listeners on relevant speech onsets, reflected by systematic amplitude reduction as compared to auditory speech only. In contrast, the hierarchical latency effect that depends upon the saliency of visual content suggests a super-additive effect of visual information that may trigger motor representations of corresponding syllable production. Further, another fMRI study showed that rehearsing nonsense sequences of syllables previously presented in audiovisual modality activated the left posterior middle temporal gyrus and some fronto-parietal sensorimotor areas more strongly than when previously perceived in auditory modality only (Venezia et al., 2016). Taken together, these results contributed to show how visual information impacts both “when” and “what” predictions types in multimodal speech processing. Although relevant, these results were obtained using isolated syllables and unlikely engaged speech sampling mechanisms supported by neural entrainment, and their cointegration by a potential central coordinator during continuous perception. Using continuous multimodal speech presentation instead, Crosse et al. (2015) showed that congruent lip movements significantly increased the cortical representation of the speech envelope by improving the correlation between evoked EEG response and the original signal, as compared to speech only. Additionally, this increase of neural entrainment by congruent visual information was greater at 2–6 Hz, corresponding to prosodic-syllabic timescales. These results suggested that in continuous speech, corresponding visual information contributes to temporal predictions (“when”) by improving stimulus-driven entrainment based on the prosodic-syllabic temporal structure. Although temporal and contents predictions are tightly related to each other during multimodal speech perception, the present review article focuses on the temporal aspect in neural predictions.

How then does the brain benefit from additional temporal information processed separately without adding extra cognitive load? In continuous multimodal perception, stimulus-driven entrainment mechanisms alone may not be sufficient to support temporal predictions as corresponding auditory and visual temporal information have to be integrated together, supposedly via additional beta-based mechanisms. If so, there should be two separable mechanisms during continuous multimodal temporal predictions, and lower beta activity may be correlated to low-frequency activity supporting entrainment mechanisms. For instance, Morillon et al. (2016) behaviorally dissociated entrainment to periodic sensory streams from temporal predictions *per se* using a target detection task where they modulated both temporal regularity and spectral dimensions in auditory tone sequences. They found that predictability of spectral context (i.e., target spectrum identical or not to the contextual noise of the tone sequence) increased the benefits of temporal predictability on target detection (i.e., synchronous vs. asynchronous sequences). In contrast, the effect of temporal regularity canceled out when a spectral prediction mismatch occurred, suggesting a hierarchy of predictions built on the perceptual context and increasing entrainment in sensory cortices. Further, Morillon and Baillet (2017) designed an experiment in which participants had to discriminate a tone sequence in phase with a reference beat from a distracting beat. This task required entrainment to an auditory stream, and the generation of internal predictions to isolate the target tone sequence. Results showed that the first mechanism relied on a delta entrainment to the auditory beat, while the second relied on cross-frequency coupling with beta (18–24 Hz) bursting at the delta rate in the left motor cortex. The authors suggested that the motor system modulates neural resource allocation via beta-based feedback to maximize delta phase entrainment in the auditory cortices, supporting the idea of contextual predictive filtering that modulates sensory entrainment. In other words, if a temporally predictable signal occurs in a multidimensional context, one dimension can modulate the temporal prediction generated by another dimension, potentially via beta-based controls.

Here, we hypothesized that the role of lower beta activity in unimodal temporal coding may actually extend to the formation of multimodal temporal predictions by driving the cooperation between afferent sensory copies from specialized areas during speech perception. We defined the term “afferent copies” as the representations of the rhythmic prosodic-syllabic speech structure extracted by the entrainment mechanisms in the auditory and visual sensory areas, which are sent to the motor areas to be integrated together to generate higher temporal predictions. These afferent copies distinguish from efferent copies describing beta-based feedback projections from motor to sensory areas in timing processing (Arnal, 2012). In other words, information of input periodicity generated by entrainment in specific sensory areas (i.e., auditory and visual cortices) converges toward a motor cortex hub to be integrated together via lower beta-based mechanisms. This integration of multiple prosodic-syllabic structure information from different modalities allows generating higher temporal prediction that provides descending feedback to auditory cortex to improve the sensory sampling of the incoming signal. On the one hand, beta oscillations encode visual motion and may respond to non-verbal information accompanying a speaker’s utterance (Kilner et al., 2000, 2003, 2009; Press et al., 2011; Jessen et al., 2012; Meirovitch et al., 2015). On the other hand, visual information conveys the prosodic-syllabic rhythms structuring the speech envelope and leads related auditory information with stable timing (Munhall et al., 2004; Pilling, 2009; Peelle and Davis, 2012; Wagner et al., 2014; Biau et al., 2016). The brain may benefit from an additional visual copy of the temporal speech structure, easing the generation of temporal predictions to improve multimodal speech perception. This visual copy of the temporal speech structure refers to the integration of regular relevant features in the visual modality conveyed by speaker’s body movements occurring at prosodic time scale. Much like prosodic features that impose rhythmic fluctuations of energy in the voice’s envelope (at 1–3 Hz), the speaker’s body provides equivalent structural information of the signal with typical corresponding movements (e.g., mouth and jaw’s aperture, eyebrows, nodes, beat gestures). Thus, if the primary auditory cortex neuron ensembles entrain to prosodic anchors (e.g., pitch peaks, power modulations, silences alternations, etc.), they might also entrain to prosodic-related movements in the visual cortex, providing an equivalent representation of the speech temporal structure at delta scale (1–3 Hz) extracted from accompanying visual modality. In turn, descending feedback to PAC may improve speech-driven entrainment and the quality of information transferred to secondary auditory cortices for multimodal binding (i.e., left superior temporal gyrus (STG)).

If true, beta responses have to respect at least three of the following criteria in audiovisual speech perception: first, lower beta activity must support temporal integration of both auditory and visual modalities during stimulus-driven entrainment, even when no direct motor behavior is required for a task. Second, (lower) beta activity must respond to biological motion in multimodal speech processing. As biological motion conveys equivalent information on the prosodic-syllabic temporal structure in multimodal speech, it is critical to establish that beta activity encodes temporal information conveyed in visual modality via speaker’s movements such in multimodal speech. Third, if beta-based temporal prediction modulates entrainment in the PAC, this should rely on a coupling between beta power bursts and the delta frequency. In the first place, auditory and visual prosodic structure representations generated in their respective sensory areas are integrated via the central amodal coordinator. Then, such delta-to-beta coupling would reflect descending beta-based feedback from motor cortex to auditory cortex to improve the delta phase alignment with prosodic cues in the incoming signal. In the current review, we report evidence regarding these three *sine qua none* criteria and address the concept of temporal prediction in multimodal speech.

## Lower Beta Activity Engages in Temporal Prediction Across Modalities

Recent research has addressed the role of lower beta power in unimodal stimulus-driven temporal prediction without direct motor engagement. Fujioka et al. (2009) investigated lower beta modulations (~15–20 Hz) with MEG during passive listening to regular auditory tone sequences with random omissions of a single tone. In the bilateral auditory cortices, beta power showed a characteristic pattern with a significant decrease within 200 ms after tone onset, returning to baseline before the onset of the expected following tone (i.e., “rebound”). In contrast, when a tone was missing, the expected decrease of power was not observed. Instead, beta power increased until the next tone occurred. Fujioka et al. (2012) extended these results and showed that while the decrease slope always occurred during the 200 ms post-tone onset independent of the periodicity of the tone sequences, the rebound slope increased with the periodicity of regular sequences (see also Cirelli et al., 2014 who replicated these results using EEG recordings in children and adults. However, they reported that the slope of power decrease varied as a function of the sequences’ periodicity). When the tone’s onsets were not predictable, the power rebound occurred earlier as compared to the regular conditions, suggesting that beta-based temporal prediction reflects the anticipation of an expected tone. These results confirmed the role of lower beta activity in the generation and maintenance of temporal prediction in passive listening to rhythmic auditory input (Fujioka et al., 2012). More recently, Fujioka et al. (2009) reported that beta activity also encodes the metric structure of isochronous auditory beats with a decrease of power dependent on whether beats were perceived as accented or not in auditory sequences. In another EEG study (Arnal et al., 2015), participants had to decide whether a final auditory target tone occurred on the beat of a preceding tone sequence or later in an active listening task. Results showed that correct responses were associated with greater beta power rebounds preceding the target onset in auditory areas as compared to incorrect responses, corroborating that beta engages in successful temporal predictions. In a recent study, Kononowicz and van Rijn (2015) investigated trial-to-trial beta power in EEG in self-paced estimations of duration. Participants pressed a button to initiate the onset of a trial and pressed again when they estimated that the interval lasted 2.5 s. Analyzing beta power pre- and post-onset, they showed that beta power positively correlated with the length of the produced durations. Hence, beta power at the onset of intervals was predictive of trial-by-trial fluctuations in the self-paced estimation of durations, before participants pressed the button to stop the trial. The origins of these lower beta oscillations still remain under debate but might originate in motor cortex, even when encoding purely temporal sensory events (Arnal, 2012; Arnal et al., 2015; Morillon and Baillet, 2017). For instance, Fujioka et al. (2009) found lower beta modulations in auditory cortices, but also in motor-related brain areas during passive listening to auditory rhythms (Fujioka et al., 2012, 2015). Further, the faster tone sequence rates engaged also the inferior frontal gyrus (IFG), supplementary motor area (SMA) and cerebellum, suggesting a motor beta contribution in an auditory temporal prediction task.

However, does lower beta power specifically support auditory temporal prediction, or support amodal mechanisms? In the latter case, lower beta power may also respond to visual stimuli. Recently, Keitel et al. (2017) investigated how the visual cortex responds to quasi-rhythmic visual simulations in different frequency ranges in attended flickering patches (i.e., theta, alpha, and beta bands). Results showed that oscillatory activity in the visual cortex tracked the temporal structure of a simple flickering stimulation, including lower beta power at 16–20 Hz. This suggests that beta activity could also track the quasi-periodic structure of exogenous visual stimulation, which may apply to real-life contexts such as the perception of speech-related movements conveying non-random dynamic information. In another study, Saleh et al. (2010) investigated the motor beta responses of a patient implanted with intracranial electrodes in the hand area of the primary motor cortex (MI), and presented him with sequences of five isochronous visual cues. In this experiment, the patient was asked to plan a motor response by the end of the sequence, dependent on the second or fourth relevant visual cue. Results showed that delta-phase entrainment increased until the informative visual cue (from the first to second, or from the first to the fourth visual stimulus), and dropped after the informative cue in both sequences. Moreover, 12–30 Hz beta power in the MI depicted a related pattern, increasing until the informative cue onset of the sequence occurred and then decreased until the end of the visual sequence. These results showed that motor beta oscillations encoded the temporal structure of isochronous visual cue sequences, and correlated to delta-phase entrainment, suggesting that delta activity modulated the responsiveness of beta oscillations at task-relevant cue onsets. Additionally, these results showed that beta activity could encode expected delays between the onset of the first visual cue and the onset of the informative cue, as the slope of power increase was significantly longer in the fourth vs. second cue sequences, although visual cues were physically identical. Thus, the beta oscillations originating from the MI are not limited to support motor preparation, but seem to play a role in the anticipation of relevant cues which require timing processing.

In summary, lower beta activity entrains to temporally structured unimodal stimulation, fulfilling the first criterion for being an amodal coordinator supporting generic temporal prediction.

## Lower Beta Activity Responds to (Speech-Related) Biological Motion and Support Multimodal Speech Integration

Numerous studies reported beta activity in response to perception and execution of actions performed by the hands or the arms (Engel and Fries, 2010; Jenkinson and Brown, 2011; Press et al., 2011; Kilavik et al., 2013; Hosaka et al., 2016; Di Nota et al., 2017). Interestingly, there seems to be a significant overlap between the origin of responding beta activity, and the neural correlates of action perception reported in fMRI or electrophysiological studies. More precisely, the pre-motor cortex, which is classically involved in passive action perception, but also the SMA, the cerebellum and the basal ganglia (di Pellegrino et al., 1992; Rizzolatti and Craighero, 2004; Morin and Grèzes, 2008), seem to engage via beta oscillation responses as well (Kilner et al., 2009; Press et al., 2011; for reviews, see also Baker, 2007; Engel and Fries, 2010). In an MEG study, Kilner et al. (2009) presented participants with videos of an actor making various movements with his right hand and arm. The authors reported a significant decrease of power in the 15–30 Hz beta band in the left and right sensors covering the sensorimotor cortex, and this attenuation occurred contralateral to the side of the screen on which the observed movement happened. In another MEG, Press et al. (2011) compared the temporal modulations of the motor activation between the execution of a sinusoidal movement with the arm and the simple observation of a person executing it. They found an equivalent decrease of 15–30 Hz beta power in the left MI in execution and observation conditions. In contrast, beta modulations were dynamically driven by the kinematics of the movement only during action observation but not execution. These results imply that beta responses support biological motion pattern analysis, which is in line with its potential role in the integration of rhythmic speech-related movement kinematics depicting a speaker’s visual prosody in speech perception, and not only general motor preparation. In other words, beta activity might support the interpretation of goal-directed simple movements with a prosodic purpose in speech context. Although these results depicted broad motor beta responses (i.e., from 13 Hz to 30 Hz across studies), other studies narrowed evidence to the lower beta band in motion perception. For instance, an EEG study showed that 19–21 Hz beta power decreased more at bilateral centro-parietal electrodes when moving dots obeyed laws of biological motion, as compared to control motions (Meirovitch et al., 2015). Further, Avanzini et al. (2012) investigated whether the observation of different types of hand movements elicited dynamic beta power patterns of modulations in an EEG study. Independently of the type of hand movement presented, they reported common desynchronization of alpha and beta (13–25 Hz) activities occurring at the onset of the action continuing for up to 400–600 ms after the movement ended, followed by a rebound at bilateral centro-parietal electrodes. Interestingly, the modulations of the 18–25 Hz beta band were correlated with the velocity of the hand movements, coding the profile of single movements although no action was required in the task. Note that the upper 20–25 Hz beta activity responses have been shown to support other cognitive processes such as working memory or attentional orienting independent of action, although not extensively discussed in the present review article (Spitzer and Blankenburg, 2012; van Ede et al., 2011). These results show that beta activity, including the lower beta band, responds to biological motion, but what about multimodal integration in speech perception? Previous studies addressed the oscillatory activity responsiveness in multimodal speech perception by investigating low-frequency entrainment in specialized areas (i.e., auditory and visual cortex) when listeners saw an accompanying facial movement of the speaker. In audiovisual speech, Park et al. (2016) investigated the coherence between oscillatory brain activity of a listener and a speaker’s lip movements. They found significant entrainment in the visual cortex correlated with lower frequency bands corresponding to the frequency spectrum of the lip signal (~4 Hz). Moreover, auditory areas were entrained by both auditory and lip movements while performance in audiovisual incongruent conditions was significantly decreased. These results show that non-verbal information modulates temporal prediction in audiovisual speech. Although this study did not report direct beta modulations, its results suggest that oscillatory activity related to a dominant speech rhythm (i.e., syllabic rate at 4 Hz) responds to non-verbal information (i.e., lip movements). Other studies focused on the role of beta activity in (speech) multimodal integration, which might be fundamental in multimodal temporal predictions (Keil et al., 2012; Schepers et al., 2013; Roa Romero et al., 2015). Schepers et al. (2013) presented isolated syllables in audiovisual vs. audio only conditions and looked at the spectro-temporal dynamics in the beta band during audiovisual speech processing. They found greater beta power suppression (16–32 Hz) after an auditory onset for the audiovisual compared to the auditory only condition in the Superior Temporal Sulcus. Additionally, the level of auditory noise added in some conditions diminished beta band suppression. In contrast, incongruent auditory and visual information (i.e., McGurk effect) elicited post-stimulus beta power suppression in the 10–30 Hz range at left central electrodes, reflecting increased integration difficulties (Keil et al., 2012; Roa Romero et al., 2015). Finally, a recent MEG study investigated speakers’ gesture integration in multimodal speech comprehension (Drijvers et al., 2018). The authors reported an alpha and beta power decrease as well as a gamma power increase when iconic gestures disambiguated degraded speech. More precisely, the left-lateralized suppression of beta power (15–20 Hz) was mainly located in the anterior cingulate cortex, SMA and IFG and correlated with the participant performance scores. Additionally, source analyses showed that the beta band suppression extended over a part of the motor cortex corresponding to the hand region of the MI. These results confirm left-lateralized motor beta oscillations in speech-related body movement perception during multimodal speech integration. Although not precisely related to multimodal integration, a study on semantic integration across modalities also reported coherence increase in beta (von Stein et al., 1999). When presenting objects in different sensory modalities (pictorial representation, written word or spoken word), they reported significant increase of coherence between temporal and parietal electrodes in the 13–18 Hz beta band as compared to rest. These results also support an amodal role of beta activity across modalities. Finally, we acknowledge that beta oscillations may not exclusively support temporal processing but other aspects in speech integration like phonological processing. A recent EEG study investigated the oscillatory correlates supporting distinct levels of analyses in continuous auditory speech processing (Mai et al., 2016). The authors presented participants with real-word sentences and sentences composed of pseudo-words (i.e., speech conditions), or backward versions of the two previous conditions (i.e., non-speech) in order to manipulate phonological and syntactic/semantic structure. They reported that low delta-theta frequencies respond to phonological (speech vs. non-speech) but not syntactic/semantic information (real vs. pseudo words), confirming their role in syllabic and supra-syllabic (like prosody) tracking. Additionally, the phonological manipulation elicited responses in higher frequencies, notably in the lower 20 Hz beta band while the syntactic/semantic manipulation elicited responses in the gamma band. These results suggested that lower beta oscillations play a role in phonological processing, independently from temporal processing, as the meter was the same across sentence conditions.

In summary, motor beta activity encodes visual speech-related information in multimodal speech, and more generally in multimodal integration, supporting the second criterion of a generic amodal coordinator for audiovisual temporal prediction. Further, this criterion together with the first criterion shows that there might be an interface between biological motion perception and sensory temporal coding mechanisms supported by lower motor beta activity (~20 Hz).

## Delta-to-Beta Coupling Supports Temporal Prediction Feedback for Entrainment to Inputs

How beta-based temporal predictions improve sensory processing should be reflected by correlated activities between low frequencies entrained to prosodic structure in the signal (i.e., 1–3 Hz delta) and lower beta oscillations. The particular role of delta activity in (multimodal) temporal predictions has been evidenced in recent studies (Kösem and van Wassenhove, 2012; Kösem et al., 2014). Indeed, the delta oscillations may provide an internal clock, and their phase alignment with external sensory inputs supports the encoding of their temporal structure. Thus, related modulations of activity between delta and lower beta oscillations may suggest active interactions between motor functions and sensory processing during temporal predictions.

Behaviorally, Morillon et al. (2014) investigated the contribution of motor activity with finger tapping on the temporal extraction of auditory tone sequences in noisy conditions. The authors presented a priming sequence of four tones at a certain pitch and participants had to discriminate the following target tones delivered on-beat from interleaved distractors to decide if the average target tone’s pitch was lower or higher than the priming ones. The perception of the target tones was greater when participants finger-tapped on the beat than when they listened to the whole sequence only. Crucially, the sensory gain obtained through motor engagement decreased when a predictable tone was affected by jittering and disappeared when participants were instructed to finger-tap in anti-phase. These results show that the motor system increases the sampling of isochronous auditory input during temporal predictions (Morillon et al., 2014, 2015). Previous MEG/EEG studies hypothesized and evidenced beta activity engagement from auditory and motor cortices in temporal predictions (Arnal, 2012; Fujioka et al., 2012, 2015; Arnal et al., 2015). For instance, Fujioka et al. (2012) examined the brain areas showing temporal correlations between beta modulations and auditory tone sequences in passive listening. First, time-frequency analyses replicated the beta (20–22 Hz) power patterns in the medial parts of bilateral PAC reported previously. Second, they explored the time courses of event-related changes in beta phase coherence in the bilateral auditory cortex A1, bilateral sensorimotor cortex SM1 and the SMA. The authors reported periodic patterns of beta coherence modulated by auditory stimuli and reflecting the sequences’ rates. However, the observed modulations of beta coherence were different between the responding areas. For instance, the beta phase coherence in the right and left primary auditory cortex areas decreased following stimulus onset, while it increased in the sensorimotor and SMA areas after stimulus onset. These results, although exploratory, suggest that isochronous auditory processing engages beta oscillatory synchronization both in auditory and motor networks as a function of rate (i.e., sequence’s period). In an MEG study, Fujioka et al. (2015) showed that the induced beta event-related desynchronization to auditory isochronous sequences varied, depending on the placement of downbeats and upbeats (march vs. waltz). The distinct patterns of responses suggested that beta oscillations may encode the metric structure of the auditory sequences. Such endogenous specific metric processing was reflected in distinct patterns of beta modulations across large neural networks including left and right auditory cortices, but also premotor cortex, sensorimotor cortex, parietal lobe and cerebellum. Recent M/EEG studies have revealed new evidence on the cooperation between auditory areas insuring the sampling of the sensory signal and motor areas supporting temporal prediction generation via cross-frequency coupling (Arnal et al., 2015; Park et al., 2015; Keitel et al., 2018). Using intracranial EEG recording, Fontolan et al. (2014) showed a role of delta phase coupling with beta activity in speech processing, but limited to the auditory areas. When they presented auditory sentences to patients, they reported a delta-beta cross-frequency coupling in the association auditory cortex that modulated the phase of gamma activity phase in the primary auditory cortex. Potentially, these results suggested that delta-beta coupling from associative areas conveyed top-down modulations on gamma-based signal processing from the primary auditory cortex. Further, Arnal et al. (2015) reported a significant beta power nesting within the delta phase at anterior left MEG sensors, with a maximum within the 200 ms preceding the target onset for correct responses. More recently, Morillon and Baillet (2017) designed an MEG experiment in which participants had to discriminate a tone sequence in phase with a reference beat from distractors. They reported a qualitative distinction between tracking and listening operating at distinct time scales. While the first mechanism relied on the delta-theta entrainment by the auditory beat, the second relied on the cross-frequency coupling with beta (18–24 Hz) bursting at the delta rate in the left motor cortex. In other words, while sensory areas extract an auditory pace via predictive coding mechanisms, the motor system modulates neural resource allocations to maximize delta phase alignment at a tone’s onsets via beta-based feedback (Arnal and Giraud, 2012; Lakatos et al., 2008; Schroeder and Lakatos, 2009b). Park et al. (2015) showed significant top-down modulations from the frontal and motor areas on the delta activity in the left auditory cortex, using effective connectivity analyses in auditory speech perception (i.e., in quantifying the degree to which motor areas causally changed the phase of auditory areas between two experimental conditions). Further, such auditory-motor cooperation is reflected by an increase of functional connectivity between the premotor cortex and the auditory cortices in beat perception (Mauritz and Wise, 1986; Grahn and Rowe, 2009; Nelson et al., 2013).

To summarize, active processing of temporally predictable auditory signals recruits left motor and auditory cortices in parallel via a communicative loop likely mediated by top-down delta-to-beta cross-frequency coupling. Consequently, beta activity meets the third criterion to be a good candidate as central coordinator.

## What’s the Role of Lower Beta in Multimodal Speech: Discussion and Perspectives

In the previous sections, we reported evidence showing that: (1) beta activity responds to temporal prediction in auditory, visual, and audiovisual (speech) stimulation. We also discussed the fact that (2) motor beta activity responds to biological motion, including non-verbal information in multimodal speech. Further there seems to be a functional overlap between biological motion perception and (sensory) temporal prediction in the lower beta band (~20 Hz). Lastly, (3) delta-to-beta cross-frequency coupling supports distant communication between (left) motor and auditory cortices when listening to speech. Meeting these three criteria, we hypothesize that lower beta plays the role of an amodal central organizer that coordinates afferent copies from visual and auditory areas in multimodal temporal prediction during speech perception. If so, a speaker’s speech-related movements (i.e., face and body) may provide a redundant copy of the temporal structure of the speech envelope at different timescales (e.g., syllabic and prosodic).

**Figure 1:**
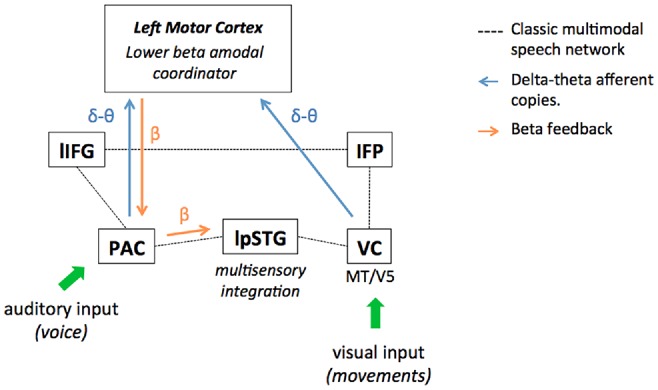
Multimodal speech perception improvement by non-verbal information in the lower motor beta oscillatory framework. The figure represents the classic left-lateralized audiovisual speech perception network and its interactions with the left motor areas in a lower beta oscillation framework. Visual and auditory sensory input is processed separately in the modality-specific visual (VC) and primary auditory cortices (PACs). Then, sampled auditory and visual input reach the secondary auditory cortex the left post superior temporal gyrus (lpSTG), where multimodal integration mechanisms engage in speech perception (which may extend to the left Inferior Parietal lobule (IFP) considered as a multisensory integration site in audiovisual speech). Finally, multimodal information is conveyed to the left inferior frontal gyrus (lIFG) for semantic integration and multimodal semantic binding. In the oscillatory framework, the visual cortex tracks non-verbal information translating the speech envelope structure conveyed at different time scales: mouth and jaw apertures convey syllabic information at theta rate (~4 Hz), while other body parts reflect the prosodic features of the speech envelope at 1–3 Hz delta rate (i.e., pitch accents, amplitude, duration, silences, etc.). In the primary auditory cortex, the delta and theta activities track the prosodic and syllabic structures composing the speech envelope from the auditory signal via phase entrainment mechanisms. While processed sensory inputs are transferred to the left STG for multimodal integration, we hypothesize that delta-theta afferent copies from primary areas are also sent to the central amodal coordinator in the motor cortex. These delta-theta afferent copies convey online information on the spectro-temporal structure of the multimodal speech (e.g., cadence, rhythm) and may facilitate the elaboration of neural top-down temporal predictions. Crucially, visual input feeds the motor cortex first, due to the natural precedence of visual over auditory onsets in audiovisual speech and prepares the incoming of redundant delta-theta information from auditory cortex. In the motor cortex, oscillatory stimulus driven input reception allows the generation of temporal predictions supported by lower beta activity. In return, beta power supports feedback to the primary auditory areas (often reported as delta-to-beta cross-frequency coupling), optimizing online its activity for the incoming speech. In theory, if auditory entrainment is improved by the beta feedback in the primary auditory cortex, this should improve the quality of information conveyed to the secondary auditory cortex as well, and then ensure better multimodal integration of the incoming speech. All in all, non-verbal information may boost the generation of lower beta-based temporal predictions by providing an additional copy of the delta-theta temporal structures of speech in the visual modality.

Non-verbal information indeed translates the slow temporal structure of speech with a stable precedence (van Wassenhove et al., 2005; Chandrasekaran et al., 2009; Biau and Soto-Faraco, 2013; Biau et al., 2015): on the one hand, the syllabic rate is reflected by the corresponding mouth aperture and jaw constraints at theta frequency (Hickok and Poeppel, 2007; Giraud and Poeppel, 2012; Peelle and Davis, 2012). On the other hand, a speaker’s prosody (i.e., modulations of amplitude, duration, and pitch accents in the speech envelope) occurs at a slower 1–3 Hz delta rate and correlates also with body movements (Munhall et al., 2004; Krahmer and Swerts, 2007; Wagner et al., 2014; Biau et al., 2016, 2017). Interestingly, speech-related motion perception has been shown to activate the speech network as well (Macaluso et al., 2004; Skipper, 2014; Biau et al., 2016). Recently, Pavlova et al. (2017) investigated the neural network of biological motion perception with moving dot presentations in an fMRI study. They reported BOLD responses in the occipital cortices, the parietal and frontal cortices, and the left fusiform gyrus, which overlaps with the multimodal speech network (Campbell, 2008; Jansma et al., 2014; Skipper, 2014). In the visual cortex, the sampling of visual information through biological motion perception may involve the bilateral MT/V5 complex as it responds more greatly to intact biological motion relative to scrambled motion, as measured by fMRI (see Peuskens et al., 2005 for example). Considering the classic audiovisual speech networks (see Calvert et al., 2000; Callan et al., 2004; Macaluso et al., 2004; Meyer et al., 2004; Campbell, 2008; Nath and Beauchamp, 2012; Jansma et al., 2014), we illustrate where non-verbal information potentially contributes to improving audiovisual speech perception by providing additional bottom-up representations of temporal structure conveyed by delta-theta activity (Figure 1). Speech-related motion may provide an additional delta-theta copy of the temporal structure via visual entrainment, preceding the auditory equivalent copy from the primary auditory cortex. The lower-frequency temporal representations across modalities would then feed a central amodal beta prediction coordinator. In turn, this would enhance delta-to-beta coupling mechanisms and tune back the primary auditory cortex to facilitate stimulus-driven entrainment. Alternatively, accompanying body and lip movements may simply accentuate the saliency of auditory features that serve as anchors to extract the syllabic-prosodic rhythms in speech. In other words, listeners anticipate and generate better temporal predictions because the auditory temporal structure is already easier to sample in the PAC (Krahmer and Swerts, 2007; Biau et al., 2016). Either way, auditory information transferred from primary to secondary auditory cortex (i.e., left post STG, lpSTG) is qualitatively improved and the multimodal speech integration facilitated. Yet, it is not clear whether the visual delta-theta afferent copies are integrated first by broader beta motor activity through biological motion perception and then sent to the lower beta generic coordinator, or feed it directly. This would explain why studies report both higher and lower beta responses in the multimodal literature, suggesting a possible functional distinction. In other words, higher motor beta activity may take care of general biological motion perception aspects, while lower motor beta activity ensures the interface between speech-related motion integration and rhythmic features from the auditory envelope. However, such a functional distinction between higher and lower beta bands has not been clearly established yet in the literature. For instance, in the present review, we reported EEG results showing that lower beta power (19–21 Hz) recorded at bilateral centro-parietal electrodes responded also to the perception of moving dots depicting biological motion (Meirovitch et al., 2015). On the other hand, other studies reported a similar power decrease-rebound response in the higher beta band (15–30 Hz) as in the lower 12–15 Hz band during isochronous tone sequence processing (Etchell et al., 2016). Future investigations will have to show how lower and higher beta band responses support different aspects during temporal processing in multimodal speech.

In the present review, we have focused on cortical interactions but it is important to note that subcortical structures involved in temporal processing, such as the basal ganglia and the cerebellum may also play a role in multimodal temporal predictions (Kotz and Schwartze, 2010). For instance, the cerebellum has been shown to respond to the visual perception of human movements (Sokolov et al., 2012; Jack et al., 2017). One can hypothesize that the cerebellum might also track the temporal structure of speech based on body movements, as it is often associated with temporal processing in speech perception and predictive coding models (Schwartze and Kotz, 2016). Additionally, the cerebellum has been shown to engage in the manipulation (i.e., encoding and retrieval) of rhythmic sequences presented either in the auditory or visual modality (Konoike et al., 2012). Other recent works showed that 10–30 Hz beta power originates from the putamen and reflects self-generated estimations of temporal durations; greater increases of beta power correlated with longer durations between consecutive taps in a synchronization-continuation task with monkeys (Bartolo et al., 2014; Bartolo and Merchant, 2015). These results suggest a role of striatal beta oscillations from deep basal ganglia structures, generally associated with movement control, but also in temporal processing (for further review on the role of the striatum in temporal processing across modalities, see Merchant et al., 2013).

Future MEG/EEG investigations may address the visual contribution in continuous multimodal speech by looking at movement-driven entrainment, reflected by the delta-theta activity responses in occipital cortex. For instance, it would be possible to compare the power in lower beta activity dependent on the temporal alignment between non-verbal and verbal information. If our hypothesis holds up, one may expect a modulation in lower motor beta oscillations when the normal precedence of visual information does not occur in audiovisual speech, or in breaking up audiovisual temporal alignment. As non-verbal information conveys speech features at different time scales, it would be interesting to investigate which body parts are correlated specifically to delta and theta entrainment modulations by mean of time-frequency analyses. On a final note, the present review may inform rehabilitative treatment of speech disorders that often relate to temporal processing deficits as in stuttering (Etchell et al., 2015, 2016; Mersov et al., 2016) or Parkinson’s speech (Kotz and Gunter, 2015). In a recent MEG study, Etchell et al. (2016) investigated beta activity responses during the presentation of isochronous tone sequences in stuttering children compared to non-stuttering children. In non-stuttering children, they reported a lower beta modulation (12–15 Hz) characterized by a decrease of power followed by a rebound preceding the onset of the next tone, reflecting temporal processing (Fujioka et al., 2012). In the stuttering group, they found a shifted pattern of lower beta power responses, resulting in an inversed rebound-decrease power modulation time-locked to the tone onsets, as compared to the typical decrease-rebound pattern in healthy children. These results show that children with a speech production deficit also exhibit abnormal lower beta activity in rhythm processing. This suggests that stuttering may relate to a problem with temporal prediction caused by a change in lower beta activity, and supports its potential role in speech-related temporal processing. In a recent article, Etchell et al. (2015) also discussed how stuttering may actually be the consequence of dysfunctions in subcortical structures belonging to the internal timing network, reflected by deviant patterns of striatal beta oscillations originating in the putamen. However, whether timing deficits relate to deficits in modality-specific entrainment or an amodal beta-based temporal prediction remains to be further specified. In the former case, providing patients with additional visual prosodic structure via a speaker’s body movements may help compensating signal-sampling deficits in the auditory modality, and still provide the lower beta coordinator with enough delta information to correctly generate temporal predictions. In the latter case, adding visual prosodic structure would not significantly improve speech perception, as the deficit arises later, when auditory and visual prosodic information is integrated with the beta-based coordinator.

## Conclusion

In the current review, we addressed the role of lower motor beta activity (~20 Hz) as a central coordinator of amodal temporal predictions during multimodal speech perception. We first reported evidence on beta engagement in unimodal temporal prediction and audiovisual integration. Second, we underlined the responsiveness of lower beta activity to biological motion and non-verbal information in audiovisual speech. Third, we described delta-to-beta cross-frequency coupling between left motor areas and auditory cortices, reflecting descending modulations on auditory entrainment. Based on these three fundamental criteria (although probably not exclusive), we hypothesized that lower motor beta activity plays a central role in generic temporal predictions by driving the cooperation between converging sensory inputs via specialized areas and conveying feedback toward the auditory cortex to facilitate audiovisual speech perception.

## Glossary

### Beta Oscillations

Ensembles of neurons that fire together at a frequency of 13–30 Hz. Beta activity can be recorded using EEG or MEG and is characterized by its phase in the cycle and its amplitude (reflected by the power). In the present review, lower beta activity corresponds to the 18–22 Hz frequency range centered around 20 Hz. While it is well established that beta activity in action perception and action originates in motor cortex (although not exclusively), it is still unclear whether it also drives temporal predictions in sensation. As discussed here, the lower band of motor beta oscillations may represent a functional interface for entrainment and temporal prediction mechanisms in multimodal speech.

### Entrainment

When sensory areas respond to rhythmic stimulation, neural populations fire at the same frequency but not necessarily together in spontaneous activity. After a number of periodic repetitions, the neurons entrain their activity to the onsets of temporally predictable events to extract the temporal structure of a sensory input and improve the sampling of the incoming signal. Consequently, the neurons align their firing phase in the frequency cycle to the same onsets, which increases the coherence of overall activity when a future event occurs, Further synchronizing activity at the neural population level decreases noise (e.g., when two neurons fire at the same frequency but not at the same time), which is reflected in an increase of power in perception. Here we propose that in multimodal speech, entrainment to both body movements and verbal utterances provides two copies of the prosodic-syllabic structures (conveyed respectively at 1–3 Hz delta and 4–7 Hz theta rates) that converge toward a generic central coordinator for temporal prediction.

### Feedback

We refer to feedback as descending information conveyed by lower beta activity from left motor cortex and improving entrainment in primary auditory cortex. Beta-based feedback on entrainment is likely supported by delta-to-beta cross-frequency coupling in motor and auditory cortices, reflected in beta bursts occurring at a delta rate.

### Speech Rhythms

Continuous speech can be decomposed into different perceptual units occurring in distinct frequency ranges. The first one corresponds to speech envelope modulations and correlates with the syllabic rate that conveys information at 4–7 Hz (theta). The second dominant speech rhythm reflects regular modulations in the utterance contour of a speaker’s prosody. The prosodic modulations of supra-segmental features of speech (such as pitch peaks, amplitude, durations, etc.) convey information at 1–3 Hz delta rate. Finally, additional acoustic information occurs at higher 30–50 Hz frequency range (gamma band) and reflects more subtle phonemic features such as fundamental formant variations.

### Non-verbal Information

We use the term non-verbal information to define all types of body movements produced by a speaker and that potentially translate auditory features at different time scales. Lip movements, mouth aperture, and jaw constraints reflect sound production and occur at a syllabic rate (~4 Hz). In contrast, simple nods of other body parts (e.g., eyebrows, shoulders, and head) translate a speaker’s voice modulations and convey information at a prosodic rate (1–3 Hz). A speaker’s gestures also accompany speech prosody with rapid flicks that align with a speaker’s prosody.

## Author Contributions

EB and SK prepared the manuscript draft. All authors approved the final manuscript.

## Conflict of Interest Statement

The authors declare that the research was conducted in the absence of any commercial or financial relationships that could be construed as a potential conflict of interest.

## References

[B1] Abrams D. A. Nicol T. Zecker S. Kraus N. (2008). Right-hemisphere auditory cortex is dominant for coding syllable patterns in speech. J. Neurosci. 28, 3958–3965. 10.1523/jneurosci.0187-08.2008 18400895 PMC2713056

[B2] Ahissar E. Nagarajan S. Ahissar M. Protopapas A. Mahncke H. Merzenich M. M. (2001). Speech comprehension is correlated with temporal response patterns recorded from auditory cortex. Proc. Natl. Acad. Sci. U S A 98, 13367–13372. 10.1073/pnas.201400998 11698688 PMC60877

[B6] Arnal L. H. (2012). Predicting “when”: using the motor system’s beta-band oscillations. Front. Hum. Neurosci. 6:225. 10.3389/fnhum.2012.00225 22876228 PMC3410664

[B3] Arnal L. H. Doelling K. B. Poeppel D. (2015). Delta-beta coupled oscillations underlie temporal prediction accuracy. Cereb. Cortex 25, 3077–3085. 10.1093/cercor/bhu103 24846147 PMC4537446

[B4] Arnal L. H. Giraud A.-L. (2012). Cortical oscillations and sensory predictions. Trends Cogn. Sci. 16, 390–398. 10.1016/j.tics.2012.05.003 22682813

[B5] Arnal L. H. Morillon B. Kell C. A. Giraud A.-L. (2009). Dual neural routing of visual facilitation in speech processing. J. Neurosci. 29, 13445–13453. 10.1523/jneurosci.3194-09.2009 19864557 PMC6665008

[B7] Avanzini P. Fabbri-Destro M. Dalla Volta R. Daprati E. Rizzolatti G. Cantalupo G. (2012). The dynamics of sensorimotor cortical oscillations during the observation of hand movements: an EEG study. PLoS One 7:e37534. 10.1371/journal.pone.0037534 22624046 PMC3356327

[B8] Baart M. Stekelenburg J. J. Vroomen J. (2014). Electrophysiological evidence for speech-specific audiovisual integration. Neuropsychologia 53, 115–121. 10.1016/j.neuropsychologia.2013.11.011 24291340

[B9] Baker S. N. (2007). Oscillatory interactions between sensorimotor cortex and the periphery. Curr. Opin. Neurobiol. 17, 649–655. 10.1016/j.conb.2008.01.007 18339546 PMC2428102

[B10] Bartolo R. Merchant H. (2015). β oscillations are linked to the initiation of sensory-cued movement sequences and the internal guidance of regular tapping in the monkey. J. Neurosci. 35, 4635–4640. 10.1523/JNEUROSCI.4570-14.2015 25788680 PMC6605135

[B11] Bartolo R. Prado L. Merchant H. (2014). Information processing in the primate basal ganglia during sensory-guided and internally driven rhythmic tapping. J. Neurosci. 34, 3910–3923. 10.1523/jneurosci.2679-13.2014 24623769 PMC6705277

[B12] Biau E. Fromont L. A. Soto-Faraco S. (2017). Beat gestures and syntactic parsing: an ERP study. Lang. Learn. 68, 102–126. 10.1111/lang.12257

[B13] Biau E. Morís Fernández L. Holle H. Avila C. Soto-Faraco S. (2016). Hand gestures as visual prosody: BOLD responses to audio-visual alignment are modulated by the communicative nature of the stimuli. Neuroimage 132, 129–137. 10.1016/j.neuroimage.2016.02.018 26892858

[B14] Biau E. Soto-Faraco S. (2013). Beat gestures modulate auditory integration in speech perception. Brain Lang. 124, 143–152. 10.1016/j.bandl.2012.10.008 23333667

[B15] Biau E. Torralba M. Fuentemilla L. de Diego Balaguer R. Soto-Faraco S. (2015). Speaker’s hand gestures modulate speech perception through phase resetting of ongoing neural oscillations. Cortex 68, 76–85. 10.1016/j.cortex.2014.11.018 25595613

[B16] Bourguignon M. De Tiège X. de Beeck M. O. Ligot N. Paquier P. Van Bogaert P.. (2013). The pace of prosodic phrasing couples the listener’s cortex to the reader’s voice. Hum. Brain Mapp. 34, 314–326. 10.1002/hbm.21442 22392861 PMC6869855

[B17] Callan D. E. Jones J. A. Callan A. M. Akahane-Yamada R. (2004). Phonetic perceptual identification by native- and second-language speakers differentially activates brain regions involved with acoustic phonetic processing and those involved with articulatory-auditory/orosensory internal models. Neuroimage 22, 1182–1194. 10.1016/j.neuroimage.2004.03.006 15219590

[B18] Calvert G. A. Campbell R. Brammer M. J. (2000). Evidence from functional magnetic resonance imaging of crossmodal binding in the human heteromodal cortex. Curr. Biol. 10, 649–657. 10.1016/s0960-9822(00)00513-3 10837246

[B19] Campbell R. (2008). The processing of audio-visual speech: empirical and neural bases. Philos. Trans. R. Soc. Lond. B Biol. Sci. 363, 1001–1010. 10.1098/rstb.2007.2155 17827105 PMC2606792

[B20] Chandrasekaran C. Trubanova A. Stillittano S. Caplier A. Ghazanfar A. A. (2009). The natural statistics of audiovisual speech. PLoS Comput. Biol. 5:e1000436. 10.1371/journal.pcbi.1000436 19609344 PMC2700967

[B21] Cirelli L. K. Bosnyak D. Manning F. C. Spinelli C. Marie C. Fujioka T.. (2014). Beat-induced fluctuations in auditory cortical beta-band activity: using EEG to measure age-related changes. Front. Psychol. 5:742. 10.3389/fpsyg.2014.00742 25071691 PMC4093753

[B22] Crosse M. J. Butler J. S. Lalor E. C. (2015). Congruent visual speech enhances cortical entrainment to continuous auditory speech in noise-free conditions. J. Neurosci. 35, 14195–14204. 10.1523/jneurosci.1829-15.2015 26490860 PMC6605423

[B25] Ding N. Melloni L. Zhang H. Tian X. Poeppel D. (2016). Cortical tracking of hierarchical linguistic structures in connected speech. Nat. Neurosci. 19, 158–164. 10.1038/nn.4186 26642090 PMC4809195

[B23] Di Nota P. M. Chartrand J. M. Levkov G. R. Montefusco-Siegmund R. DeSouza J. F. X. (2017). Experience-dependent modulation of alpha and beta during action observation and motor imagery. BMC Neurosci. 18:28. 10.1186/s12868-017-0349-0 28264664 PMC5340035

[B24] di Pellegrino G. Fadiga L. Fogassi L. Gallese V. Rizzolatti G. (1992). Understanding motor events: a neurophysiological study. Exp. Brain Res. 91, 176–180. 10.1007/bf00230027 1301372

[B26] Drijvers L. Özyürek A. Jensen O. (2018). Hearing and seeing meaning in noise: alpha, beta and gamma oscillations predict gestural enhancement of degraded speech comprehension. Hum. Brain Mapp. 39, 2075–2087. 10.1002/hbm.23987 29380945 PMC5947738

[B27] Engel A. K. Fries P. (2010). Beta-band oscillations-signalling the status quo? Curr. Opin. Neurobiol. 20, 156–165. 10.1016/j.conb.2010.02.015 20359884

[B28] Etchell A. C. Johnson B. W. Sowman P. F. (2015). Beta oscillations, timing, and stuttering. Front. Hum. Neurosci. 8:1036. 10.3389/fnhum.2014.01036 25601832 PMC4283545

[B29] Etchell A. C. Ryan M. Martin E. Johnson B. W. Sowman P. F. (2016). Abnormal time course of low beta modulation in non-fluent preschool children: a magnetoencephalographic study of rhythm tracking. Neuroimage 125, 953–963. 10.1016/j.neuroimage.2015.10.086 26545455

[B30] Fontolan L. Morillon B. Liegeois-Chauvel C. Giraud A.-L. (2014). The contribution of frequency-specific activity to hierarchical information processing in the human auditory cortex. Nat. Commun. 5:4694. 10.1038/ncomms5694 25178489 PMC4164774

[B31] Fujioka T. Ross B. Trainor L. J. (2015). Beta-band oscillations represent auditory beat and its metrical hierarchy in perception and imagery. J. Neurosci. 35, 15187–15198. 10.1523/jneurosci.2397-15.2015 26558788 PMC6605356

[B32] Fujioka T. Trainor L. J. Large E. W. Ross B. (2009). Beta and gamma rhythms in human auditory cortex during musical beat processing. Ann. N Y Acad. Sci. 1169, 89–92. 10.1111/j.1749-6632.2009.04779.x 19673759

[B33] Fujioka T. Trainor L. J. Large E. W. Ross B. (2012). Internalized timing of isochronous sounds is represented in neuromagnetic β oscillations. J. Neurosci. 32, 1791–1802. 10.1523/jneurosci.4107-11.2012 22302818 PMC6703342

[B34] Giraud A.-L. Poeppel D. (2012). Cortical oscillations and speech processing: emerging computational principles and operations. Nat. Neurosci. 15, 511–517. 10.1038/nn.3063 22426255 PMC4461038

[B35] Grahn J. A. Rowe J. B. (2009). Feeling the beat: premotor and striatal interactions in musicians and nonmusicians during beat perception. J. Neurosci. 29, 7540–7548. 10.1523/jneurosci.2018-08.2009 19515922 PMC2702750

[B36] Gross J. Hoogenboom N. Thut G. Schyns P. Panzeri S. Belin P.. (2013). Speech rhythms and multiplexed oscillatory sensory coding in the human brain. PLoS Biol. 11:e1001752. 10.1371/journal.pbio.1001752 24391472 PMC3876971

[B37] Hickok G. Poeppel D. (2007). The cortical organization of speech processing. Nat. Rev. Neurosci. 8, 393–402. 10.1038/nrn2113 17431404

[B38] Hosaka R. Nakajima T. Aihara K. Yamaguchi Y. Mushiake H. (2016). The suppression of beta oscillations in the primate supplementary motor complex reflects a volatile state during the updating of action sequences. Cereb. Cortex 26, 3442–3452. 10.1093/cercor/bhv163 26232988

[B39] Jack A. Keifer C. M. Pelphrey K. A. (2017). Cerebellar contributions to biological motion perception in autism and typical development. Hum. Brain Mapp. 38, 1914–1932. 10.1002/hbm.23493 28150911 PMC5342927

[B40] Jansma H. Roebroeck A. Münte T. F. (2014). A network analysis of audiovisual affective speech perception. Neuroscience 256, 230–241. 10.1016/j.neuroscience.2013.10.047 24184115

[B41] Jenkinson N. Brown P. (2011). New insights into the relationship between dopamine, beta oscillations and motor function. Trends Neurosci. 34, 611–618. 10.1016/j.tins.2011.09.003 22018805

[B42] Jessen S. Obleser J. Kotz S. A. (2012). How bodies and voices interact in early emotion perception. PLoS One 7:e36070. 10.1371/journal.pone.0036070 22558332 PMC3340409

[B43] Keil J. Müller N. Ihssen N. Weisz N. (2012). On the variability of the McGurk effect: audiovisual integration depends on prestimulus brain states. Cereb. Cortex 22, 221–231. 10.1093/cercor/bhr125 21625011

[B44] Keitel A. Gross J. Kayser C. (2018). Perceptually relevant speech tracking in auditory and motor cortex reflects distinct linguistic features. PLoS Biol. 16:e2004473. 10.1371/journal.pbio.2004473 29529019 PMC5864086

[B45] Keitel C. Thut G. Gross J. (2017). Visual cortex responses reflect temporal structure of continuous quasi-rhythmic sensory stimulation. Neuroimage 146, 58–70. 10.1016/j.neuroimage.2016.11.043 27867090 PMC5312821

[B46] Kilavik B. E. Zaepffel M. Brovelli A. MacKay W. A. Riehle A. (2013). The ups and downs of beta oscillations in sensorimotor cortex. Exp. Neurol. 245, 15–26. 10.1016/j.expneurol.2012.09.014 23022918

[B47] Kilner J. M. Baker S. N. Salenius S. Hari R. Lemon R. N. (2000). Human cortical muscle coherence is directly related to specific motor parameters. J. Neurosci. 20, 8838–8845. 10.1523/jneurosci.20-23-08838.2000 11102492 PMC6773054

[B48] Kilner J. M. Marchant J. L. Frith C. D. (2009). Relationship between activity in human primary motor cortex during action observation and the mirror neuron system. PLoS One 4:e4925. 10.1371/journal.pone.0004925 19290052 PMC2654140

[B49] Kilner J. M. Salenius S. Baker S. N. Jackson A. Hari R. Lemon R. N. (2003). Task-dependent modulations of cortical oscillatory activity in human subjects during a bimanual precision grip task. Neuroimage 18, 67–73. 10.1006/nimg.2002.1322 12507444

[B50] Konoike N. Kotozaki Y. Miyachi S. Miyauchi C. M. Yomogida Y. Akimoto Y.. (2012). Rhythm information represented in the fronto-parieto-cerebellar motor system. Neuroimage 63, 328–338. 10.1016/j.neuroimage.2012.07.002 22796994

[B51] Kononowicz T. W. van Rijn H. (2015). Single trial beta oscillations index time estimation. Neuropsychologia 75, 381–389. 10.1016/j.neuropsychologia.2015.06.014 26102187

[B52] Kösem A. Gramfort A. van Wassenhove V. (2014). Encoding of event timing in the phase of neural oscillations. Neuroimage 92, 274–284. 10.1016/j.neuroimage.2014.02.010 24531044

[B53] Kösem A. van Wassenhove V. (2012). Temporal structure in audiovisual sensory selection. PLoS One 7:e40936. 10.1371/journal.pone.0040936 22829899 PMC3400621

[B54] Kotz S. A. Gunter T. C. (2015). Can rhythmic auditory cuing remediate language related deficits in Parkinson’s disease? Ann. N Y Acad. Sci. 1337, 62–68. 10.1111/nyas.12657 25773618

[B55] Kotz S. A. Schwartze M. (2010). Cortical speech processing unplugged: a timely subcortico-cortical framework. Trends Cogn. Sci. 14, 392–399. 10.1016/j.tics.2010.06.005 20655802

[B56] Krahmer E. Swerts M. (2007). The effects of visual beats on prosodic prominence: acoustic analyses, auditory perception and visual perception. J. Mem. Lang. 57, 396–414. 10.1016/j.jml.2007.06.005

[B57] Lakatos P. Karmos G. Mehta A. D. Ulbert I. Schroeder C. E. (2008). Entrainment of neuronal oscillations as a mechanism of attentional selection. Science 320, 110–113. 10.1126/science.1154735 18388295

[B58] Luo H. Poeppel D. (2007). Phase patterns of neuronal responses reliably discriminate speech in human auditory cortex. Neuron 54, 1001–1010. 10.1016/j.neuron.2007.06.004 17582338 PMC2703451

[B59] Macaluso E. George N. Dolan R. Spence C. Driver J. (2004). Spatial and temporal factors during processing of audiovisual speech: a PET study. Neuroimage 21, 725–732. 10.1016/j.neuroimage.2003.09.049 14980575

[B60] Mai G. Minett J. W. Wang W. S.-Y. (2016). Delta, theta, beta, and gamma brain oscillations index levels of auditory sentence processing. Neuroimage 133, 516–528. 10.1016/j.neuroimage.2016.02.064 26931813

[B61] Mauritz K. H. Wise S. P. (1986). Premotor cortex of the rhesus monkey: neuronal activity in anticipation of predictable environmental events. Exp. Brain Res. 61, 229–244. 10.1007/bf00239513 3948938

[B62] Meirovitch Y. Harris H. Dayan E. Arieli A. Flash T. (2015). Alpha and beta band event-related desynchronization reflects kinematic regularities. J. Neurosci. 35, 1627–1637. 10.1523/JNEUROSCI.5371-13.2015 25632138 PMC6795263

[B63] Merchant H. Harrington D. L. Meck W. H. (2013). Neural basis of the perception and estimation of time. Annu. Rev. Neurosci. 36, 313–336. 10.1146/annurev-neuro-062012-170349 23725000

[B64] Mersov A. M. Jobst C. Cheyne D. O. De Nil L. (2016). Sensorimotor oscillations prior to speech onset reflect altered motor networks in adults who stutter. Front. Hum. Neurosci. 10:443. 10.3389/fnhum.2016.00443 27642279 PMC5009120

[B65] Meyer M. Steinhauer K. Alter K. Friederici A. D. von Cramon D. Y. (2004). Brain activity varies with modulation of dynamic pitch variance in sentence melody. Brain Lang. 89, 277–289. 10.1016/s0093-934x(03)00350-x 15068910

[B66] Morillon B. Baillet S. (2017). Motor origin of temporal predictions in auditory attention. Proc. Natl. Acad. Sci. U S A 114, E8913–E8921. 10.1073/pnas.1705373114 28973923 PMC5651745

[B67] Morillon B. Hackett T. A. Kajikawa Y. Schroeder C. E. (2015). Predictive motor control of sensory dynamics in auditory active sensing. Curr. Opin. Neurobiol. 31, 230–238. 10.1016/j.conb.2014.12.005 25594376 PMC4898262

[B69] Morillon B. Schroeder C. E. Wyart V. (2014). Motor contributions to the temporal precision of auditory attention. Nat. Commun. 5:5255. 10.1038/ncomms6255 25314898 PMC4199392

[B68] Morillon B. Schroeder C. E. Wyart V. Arnal L. H. (2016). Temporal prediction in lieu of periodic stimulation. J. Neurosci. 36, 2342–2347. 10.1523/JNEUROSCI.1277-16.2016 26911682 PMC4860448

[B70] Morin O. Grèzes J. (2008). What is “mirror” in the premotor cortex? A review. Neurophysiol. Clin. 38, 189–195. 10.1016/j.neucli.2008.02.005 18539253

[B71] Munhall K. G. Jones J. A. Callan D. E. Kuratate T. Vatikiotis-Bateson E. (2004). Visual prosody and speech intelligibility: head movement improves auditory speech perception. Psychol. Sci. 15, 133–137. 10.1111/j.0963-7214.2004.01502010.x 14738521

[B72] Nath A. R. Beauchamp M. S. (2012). A neural basis for interindividual differences in the McGurk effect, a multisensory speech illusion. Neuroimage 59, 781–787. 10.1016/j.neuroimage.2011.07.024 21787869 PMC3196040

[B73] Nelson A. Schneider D. M. Takatoh J. Sakurai K. Wang F. Mooney R. (2013). A circuit for motor cortical modulation of auditory cortical activity. J. Neurosci. 33, 14342–14353. 10.1523/JNEUROSCI.2275-13.2013 24005287 PMC3761045

[B74] Nourski K. V. Reale R. A. Oya H. Kawasaki H. Kovach C. K. Chen H.. (2009). Temporal envelope of time-compressed speech represented in the human auditory cortex. J. Neurosci. 29, 15564–15574. 10.1523/JNEUROSCI.3065-09.2009 20007480 PMC2851231

[B75] Park H. Ince R. A. A. Schyns P. G. Thut G. Gross J. (2015). Frontal top-down signals increase coupling of auditory low-frequency oscillations to continuous speech in human listeners. Curr. Biol. 25, 1649–1653. 10.1016/j.cub.2015.04.049 26028433 PMC4503802

[B76] Park H. Kayser C. Thut G. Gross J. (2016). Lip movements entrain the observers’ low-frequency brain oscillations to facilitate speech intelligibility. Elife 5:e14521. 10.7554/elife.14521 27146891 PMC4900800

[B77] Pavlova M. A. Erb M. Hagberg G. E. Loureiro J. Sokolov A. N. Scheffler K. (2017). “Wrong way up”: temporal and spatial dynamics of the networks for body motion processing at 9.4 T. Cereb. Cortex 27, 5318–5330. 10.1093/cercor/bhx151 28981613

[B78] Peelle J. E. Davis M. H. (2012). Neural oscillations carry speech rhythm through to comprehension. Front. Psychol. 3:320. 10.3389/fpsyg.2012.00320 22973251 PMC3434440

[B79] Peelle J. E. Sommers M. S. (2015). Prediction and constraint in audiovisual speech perception. Cortex 68, 169–181. 10.1016/j.cortex.2015.03.006 25890390 PMC4475441

[B80] Peuskens H. Vanrie J. Verfaillie K. Orban G. (2005). Specificity of regions processing biological motion. Eur. J. Neurosci. 21, 2864–2875. 10.1111/j.1460-9568.2005.04106.x 15926934

[B81] Pilling M. (2009). Auditory event-related potentials (ERPs) in audiovisual speech perception. J. Speech Lang. Hear. Res. 52, 1073–1081. 10.1044/1092-4388(2009/07-0276) 19641083

[B82] Press C. Cook J. Blakemore S.-J. Kilner J. (2011). Dynamic modulation of human motor activity when observing actions. J. Neurosci. 31, 2792–2800. 10.1523/JNEUROSCI.1595-10.2011 21414901 PMC3398132

[B83] Rizzolatti G. Craighero L. (2004). The mirror-neuron system. Annu. Rev. Neurosci. 27, 169–192. 10.1146/annurev.neuro.27.070203.144230 15217330

[B84] Roa Romero Y. Senkowski D. Keil J. (2015). Early and late beta-band power reflect audiovisual perception in the McGurk illusion. J. Neurophysiol. 113, 2342–2350. 10.1152/jn.00783.2014 25568160 PMC4416591

[B85] Saleh M. Reimer J. Penn R. Ojakangas C. L. Hatsopoulos N. G. (2010). Fast and slow oscillations in human primary motor cortex predict oncoming behaviorally relevant cues. Neuron 65, 461–471. 10.1016/j.neuron.2010.02.001 20188651 PMC3199221

[B86] Schepers I. M. Schneider T. R. Hipp J. F. Engel A. K. Senkowski D. (2013). Noise alters beta-band activity in superior temporal cortex during audiovisual speech processing. Neuroimage 70, 101–112. 10.1016/j.neuroimage.2012.11.066 23274182

[B88] Schroeder C. E. Lakatos P. (2009a). Low-frequency neuronal oscillations as instruments of sensory selection. Trends Neurosci. 32, 9–18. 10.1016/j.tins.2008.09.012 19012975 PMC2990947

[B89] Schroeder C. E. Lakatos P. (2009b). The gamma oscillation: master or slave? Brain Topogr. 22, 24–26. 10.1007/s10548-009-0080-y 19205863 PMC2989849

[B87] Schroeder C. E. Lakatos P. Kajikawa Y. Partan S. Puce A. (2008). Neuronal oscillations and visual amplification of speech. Trends Cogn. Sci. 12, 106–113. 10.1016/j.tics.2008.01.002 18280772 PMC3987824

[B90] Schwartze M. Kotz S. A. (2016). Contributions of cerebellar event-based temporal processing and preparatory function to speech perception. Brain Lang. 161, 28–32. 10.1016/j.bandl.2015.08.005 26362972

[B91] Skipper J. I. (2014). Echoes of the spoken past: how auditory cortex hears context during speech perception. Philos. Trans. R. Soc. Lond. B Biol. Sci. 369:20130297. 10.1098/rstb.2013.0297 25092665 PMC4123676

[B92] Sokolov A. A. Erb M. Gharabaghi A. Grodd W. Tatagiba M. S. Pavlova M. A. (2012). Biological motion processing: the left cerebellum communicates with the right superior temporal sulcus. Neuroimage 59, 2824–2830. 10.1016/j.neuroimage.2011.08.039 22019860

[B93] Spitzer B. Blankenburg F. (2012). Supramodal parametric working memory processing in humans. J. Neurosci. 32, 3287–3295. 10.1523/JNEUROSCI.5280-11.2012 22399750 PMC6621033

[B94] Stekelenburg J. J. Vroomen J. (2007). Neural correlates of multisensory integration of ecologically valid audiovisual events. J. Cogn. Neurosci. 19, 1964–1973. 10.1162/jocn.2007.19.12.1964 17892381

[B95] van Ede F. de Lange F. Jensen O. Maris E. (2011). Orienting attention to an upcoming tactile event involves a spatially and temporally specific modulation of sensorimotor alpha- and beta-band oscillations. J. Neurosci. 31, 2016–2024. 10.1523/JNEUROSCI.5630-10.2011 21307240 PMC6633042

[B96] van Wassenhove V. Grant K. W. Poeppel D. (2005). Visual speech speeds up the neural processing of auditory speech. Proc. Natl. Acad. Sci. U S A 102, 1181–1186. 10.1073/pnas.0408949102 15647358 PMC545853

[B97] Venezia J. H. Fillmore P. Matchin W. Lisette Isenberg A. Hickok G. Fridriksson J. (2016). Perception drives production across sensory modalities: a network for sensorimotor integration of visual speech. Neuroimage 126, 196–207. 10.1016/j.neuroimage.2015.11.038 26608242 PMC4733636

[B98] von Stein A. Rappelsberger P. Sarnthein J. Petsche H. (1999). Synchronization between temporal and parietal cortex during multimodal object processing in man. Cereb. Cortex 9, 137–150. 10.1093/cercor/9.2.137 10220226

[B99] Vroomen J. Stekelenburg J. J. (2010). Visual anticipatory information modulates multisensory interactions of artificial audiovisual stimuli. J. Cogn. Neurosci. 22, 1583–1596. 10.1162/jocn.2009.21308 19583474

[B100] Wagner P. Malisz Z. Kopp S. (2014). Gesture and speech in interaction: an overview. Speech Commun. 57, 209–232. 10.1016/j.specom.2013.09.008

[B101] Zoefel B. VanRullen R. (2016). EEG oscillations entrain their phase to high-level features of speech sound. Neuroimage 124, 16–23. 10.1016/j.neuroimage.2015.08.054 26341026

